# Levels of Faecal Calprotectin and Magnetic Resonance Enterocolonography Correlate with Severity of Small Bowel Crohn’s Disease: A Retrospective Cohort Study

**DOI:** 10.1038/s41598-017-02111-6

**Published:** 2017-05-16

**Authors:** Lei Ye, Wei Cheng, Bi-qin Chen, Xing Lan, Shao-dong Wang, Xiao-chen Wu, Wei Huang, Fang-yu Wang

**Affiliations:** 1 Department of Gastroenterology and Hepatology, Jinling Hospital, Medical School of Nanjing University, 305 Zhongshan East Road, Nanjing, 210002 Jiangsu Province China; 2 Department of Gastroenterology and Hepatology, Jinling Hospital, Clinical Medical School of Southern Medical University, Nanjing, 210002 China; 3 School of Medical Imaging, Xuzhou Medical University, Xuzhou, 221004 China; 4 Department of Medical Imaging, Jinling Hospital, Medical School of Nanjing University, 305 Zhongshan East Road, Nanjing, 210002 Jiangsu Province China

## Abstract

Few studies have evaluated the usefulness of fecal calprotectin (FC) or magnetic resonance enterography (MRE) in diagnosing active Crohn’s disease (CD) of the small bowel. In the study, we investigated the reliability of FC and MRE in assessing the activity of ileal CD and further explored the relationship between levels of FC and MRE scores. A total of 221 patients were diagnosed with ileal or ileo-colitis CD in our department between July 2012 and October 2016. The global magnetic resonance index of activity (MaRIA) correlated with the simple endoscopic score for CD (SES-CD) (r = 0.527, P = 0.005). When analysed segment-by-segment, a significant correlation was still observed (r = 0.590, P < 0.001). The SES-CD correlated closest with FC (r = 0.503), followed by CRP (r = 0.461), ESR (0.377) and the CDAI (r = 0.320). In receiver operating characteristic (ROC) analyses, the FC cut-off value of mucosal healing was 213.1 μg/g, with 76.1% sensitivity and 66.7% specificity. As for MaRIA, a cut-off value of 6.8 for each segment provided a sensitivity of 100% and a specificity of 79.2%. No agreement between MaRIA and FC levels was found. In conclusion, a combination of FC levels and MaRIA could be effective in monitoring mucosal activity in patients with small bowel CD.

## Introduction

Crohn’s disease (CD) is characterized by chronic inflammation in the gut and periods of remission and intermittent relapses. The last decade has witnessed a breakthrough in the therapeutic management of CD. The introduction of anti-tumour necrosis factor (TNF) agents such as infliximab (IFX) and adalimumab (ADA) has enormously reduced CD-related hospitalizations and surgeries^1,2^. Consequently, the therapeutic paradigm has shifted from symptom-based (by measuring the Crohn’s disease activity index (CDAI)) to mucosal healing^3–5^. Since CD lesions can involve the whole gastrointestinal tract, both the large and small bowel must be accurately assessed to determine the extent and severity of the disease. It has been reported that 4% to 65% of CD patients may have small bowel lesions^6,7^, but conventional ileocolonoscopy (ICS) cannot detect disease in the deep small bowel. Balloon-assisted enteroscopy (BAE), a new technique, seems to be feasible and superior for a concise assessment of the mucosa^8,9^. Nevertheless, the reach of the scope and the detection of small aphthae rely on the skill of the performer. Additionally, BAE would be problematic when detecting stenosis or intestinal adhesions in CD.

In recent years, interest in cross-sectional modalities has emerged rapidly. Computed tomography enterography (CTE) and magnetic resonance enterography (MRE) are experiencing the largest expansions^10–13^. Recently, it has been reported that computed tomography could significantly increase diagnostic radiation, which may contribute to the development of malignancy, considering that patients with inflammatory bowel disease are at a relative high risk of developing gut carcinoma^14,15^. Characterized by a high soft-tissue contrast, a lack of ionizing radiation and a low incidence of adverse events, MRE is more suitable for widespread acceptance^16^.

Faecal calprotectin (FC), an important cytosolic protein released from neutrophils, is stable for a week at room temperature^17,18^. Numerous studies have demonstrated that FC levels demonstrate a good correlation with intestinal inflammation^5,19–21^. However, few studies have evaluated the relationship between FC levels and mucosal lesions in small bowel CD.

The majority of published MRI studies on CD focus on the correlation between various signs and endoscopy findings. Although clinically significant, this information is not very feasible for use in guiding therapeutic decisions. Rimola *et al*. have described a quantitative Magnetic Resonance Index of Activity (MaRIA) that is well correlated with the Crohn’s Disease Endoscopic Index of Severity (CDEIS)^12^. However, patients who were included were experiencing ICS, and small bowel CD has not been evaluated.

The aim of this study was to explore whether the levels of FC would be superior to CDAI, ESR, and CRP to distinguish endoscopically active disease in small bowel CD and to evaluate the correlation between MaRIA and endoscopy findings. We hypothesized that FC and MaRIA would be non-invasive markers to assess small bowel CD activity, and therefore, ICS or BAE would be avoided in some cases before clinical decision-making.

## Materials and Methods

### Patients

Overall, 368 patients with a suspected CD or previous diagnosis of CD were referred to the Department of Gastroenterology and Hepatology of Jinling Hospital between July 2012 and October 2016. Among them, 147 patients were investigated to assess for the presence of colonic CD. We excluded patients who had colonic CD because we focused only on small intestinal CD. Thus, 221 patients were available for the primary analysis. Patients were given the informed consent before using their data.

### Data collection

The study was approved by the Jinling Ethics Committee. Data were collected retrospectively by detailed case-note review. Detailed demographic and phenotypic data^22^, therapy data, FC levels, MRE, ICS or ileoscopy results and serum inflammatory markers were recorded. Serum C-reactive protein (CRP, normal range <10 mg/L) levels and erythrocyte sedimentation rate (ESR, normal range 0–15 mm/h) measured on admission were compared with FC levels. In our institution, FC measurement was an optional indicator for intestinal inflammation until January 2015. In all, the FC level was obtained in 213 of 221 enrolled patients. Since repeated enteroscopy is not acceptable in patients with established CD, 163 patients underwent either ICS or single-balloon endoscopy (SBE). Since 2014, as a newly established technique in Jinling Hospital, MRE has been available for CD patients. There were 73 out of 221 patients underwent MRE. As for CD patients in our department, the most common hospitalization time was within 7 days. Therefore, FC levels, enteroscopy findings and MRE findings were recorded within 1 week of serum markers when correlations were evaluated.

### FC measurement

FC was measured using a specific ELISA procedure according to the manufacturer’s instructions (Buhlmann fCAL^TM^ ELISA, Buhlmann, Switzerland). Faecal samples were diluted to 1:49. Enzyme-linked immunosorbent assay plates were read on a Spectra reader (Bio-Rad, USA) at an optical density of 450 nm. The FC levels were expressed as micrograms of FC per gram of faeces.

### Endoscopic disease activity

Ninety ICS procedures and 73 SBE procedures were performed by experienced endoscopists. The retrograde approach was used to carry out the SBE procedures. If the scope was difficulty to pass due to a stenosis, then a contrast medium was injected to ensure there was not a sharp angulation. The distance from the ileocaecal valve or the postoperative anastomosis to the deepest point was recorded.

The severity and extent of the inflammatory lesions were evaluated using the simple endoscopic score for CD (SES-CD)^23^. The small bowel was divided into 3 sections: the terminal ileum, defined as the section ≤10 cm from the ileocaecal valve or the anastomotic site; the proximal ileum, defined as the small bowel between the 10 cm from the ileocaecal valve and 300 cm from the ileocaecal valve; and the jejunum, defined as the remaining small bowel^24^. To calculate the global SES-CD, the three parts of small bowel were represented by the most severely affected part. Thus, the new “terminal ileum” substitutes for the pre-defined terminal ileum in the SES-CD. Based on the SES-CD, endoscopic remission and the severity of the mucosal inflammation were defined as follows: inactive 0–3, mild activity 4–10, moderate activity 11–19, and severe activity ≥20 points^5^.

### MRE technique

Bowel preparation was obtained through oral administration of a folium senna solution or enemas. Patients fasted for 6 hours before the MRE. All the patients were instructed to drink 2000 ml of 2.5% sorbitol solution to completely distend the bowel 1.5 hours before magnetic resonance (MR) scanning. Then, a total of 10 mg of anisodamine was administered for bowel peristalsis 5 minutes before examination. MR scanning was performed using a 3-T system (Signa Excite; GE healthCare, Milwaukee, WI, United States). All the patients were scanned routinely with a 32-channel, phased-array body coil in the supine position. The MR examination protocol and scan parameters were as follows: coronal T2-weighted single shot fast spin-echo with and without fat-suppressed sequences (TR/TE, 1480 ms/68 ms; slice thickness/spacing, 4 mm/1 mm; FOV, 42 cm × 37.8 cm; matrix, 288 × 288; bandwidth, 83.33 Hz/pixel; NEX, 0.53); axial T2-weighted propeller with fat-suppressed sequences (TR/TE, 16667 ms/75.4 ms; slice thickness/slice spacing, 4 mm/1 mm; FOV, 42 cm × 42 cm; bandwidth, 83.33 KHz; matrix, 320 × 320; NEX, 2.5); axial DWI spin echo echoplanar sequences (TR/TE, 8000 ms/48 ms; FOV, 38 cm × 30 cm; slice thickness/slice spacing, 4 mm/1 mm; matrix, 96 × 128; bandwidth, 250 kHz; number shots, 1; b value 0 and 800 s/mm^2^; NEX, 1); coronal T1-weighted LAVA-FLEX with fat-suppressed images were acquired with the breath-holding technique before and after intravenous contrast media administration (TR/TE, 4 ms/0 ms; slice thickness/interslice, 4 mm/0 mm; flip angle, 13; FOV, 42 cm × 48 cm; matrix, 272 × 192; bandwidth, 142 KHz; NEX, 1).

### MRE image analysis

Two experienced radiologists blindly evaluated the MRE findings according to the four parameters evaluated in the previous study^12^. The relative contrast enhancement (RCE) was calculated according to the following formula: RCE = [(WSI postgadolinium-WSI pregadolinium)/(WSI pregadolinium)] × 100 × (SD noise pregadolinium/SD noise postgadolinium), which has been described in the study by Rimola *et al*.^12^. Examples of MRE alterations associated with the presence of active inflammation are shown in Figure S1.

### Statistical analysis

Statistical analyses were performed using the Statistical Package for Social Sciences (SPSS) version 18 software (SPSS Inc., Chicago, IL, USA). Continuous variables are presented as the median and interquartile range (IQR). For continuous variables with a non-parametric distribution, the differences between groups were compared using the Mann-Whitney U test, and categorical variables were compared using the chi-squared test. Receiver operating characteristic (ROC) curves were drawn to determine the best threshold of FC and MRE scores to assess the endoscopic mucosal healing of CD patients (defined as SES-CD = 0). The results are presented as the sensitivity, specificity, and positive and negative predictive values. Spearman’s rank correlation coefficient was used to assess the association between SES-CD and MaRIA, FC, ESR, CRP or CDAI. To identify predictors of mucosal severity in patients, multivariate analysis was performed using binary logistic regression analysis. MaRIA, FC, ESR, CRP and CDAI were adjusted by “Forward: LR” method of statistical program as covariates in regression model. A two-sided P value of <0.05 was considered statistically significant for all analyses. The scatter plot and bar graph were generated using GraphPad Prism 5.0 (GraphPad Software Inc., La Jolla, USA).

## Results

### Demographic and clinical data

A total of 221 patients were eligible for inclusion, 51 of whom were limited to small bowel lesions. There was a significant bias between sexes, with 76 females (34.4%) compared to 145 males (65.6%). The majority had known CD with a median disease duration of 25 months (IQR 11–72). Eighty-four patients (38%) had undergone surgery due to disease-related complications. As shown in Table 1, 19 patients had undergone surgery for small bowel lesions; 5 had ileocaecal resection, and 12 had left/right hemicolectomy. In contrast, as many as 49 out of 84 patients had undergone surgery for anal fistulas or a perianal abscess. Fourteen patients (6.3%) patients had received anti-tumour necrosis factor agents such as infliximab, and 37 patients (16.7%) were administered immunomodulators such as azathioprine.

**Table 1:** Baseline demographic variables of study population.

Female sex (%)	76 (34.4%)
Median age at diagnosis, year (IQR)	30 (23.5–44.0)
Median disease duration, month (IQR)	25 (11–72)
Disease location	
L1/L3/L4	51/170/3
Disease behavior	
B1/B2/B3	97/95/34
Smoking	
No/Ex/Yes	21/3/1/7
Perianal disease (%)	49 (22.2%)
Previous surgery (%)	84 (38%)
Surgical location	
Ileum/ileo-caecal/colon/perianal disease	19/5/12/49
Medication	
Anti-tumor necrosis factor (TNF) agents (%)	14 (6.3%)
Immunomodulator (%)	37 (16.7%)

IQR: interquartile range; Disease Location: L1, ileitis; L2, colitis; L3, ileo-colitis; L4, upper gastrointestinal involvement; Disease behavior: B1, non-stricturing, non-penetrating; B2, structuring; B3, penetrating.

Compared to patients with ileocolitis, both inflammatory marker levels and enteroscopy scores seemed to be significantly lower for patients whose intestinal lesions were confined to the small bowel (Table 2). Among the total of 221 patients, FC values were available in 213 patients, and the results showed no difference between the two subgroups. Consistent with the enteroscopy findings, the MRE findings demonstrated heavier mucosal lesions in patients with ileocolitis.

**Table 2:** Clinical and laboratory characteristics according to disease location in small bowel Crohn’s disease patients.

	Ileitis	Ileo-colitis	P value
	(N = 51)	(N = 170)	
SES-CD, N	51	112	<0.001
median (IQR)	0 (0–4)	5 (3–9)	
FC (μg/g),N	51	162	0.148
median (IQR)	254.4 (95.4–914.6)	584.7 (136.3–1081.6)	
ESR (mm/h), N	43	147	0.001
median (IQR)	12 (6–23)	20 (11–40)	
CRP (mg/L), N	51	170	0.001
median (IQR)	4.2 (0.9–15)	12.7 (2.0–44.0)	
MaRIA, N	16	57	0.002
median (IQR)	21.53 (9.6–33.4)	39.5 (20.9–63.3)	
CDAI, N	44	163	0.052
median (IQR)	129 (83.5–184.8)	173 (87–253)	

IQR, interquartile range; SES-CD, simple endoscopic score for Crohn’s disease; FC, faecal calprotectin; ESR, erythrocyte sedimentation rate; CRP, C-reactive protein; MaRIA, Magnetic resonance index of activity; CDAI, Crohn’s disease activity index.

### Relationships between MRE findings and enteroscopy findings

**Figure 1:**
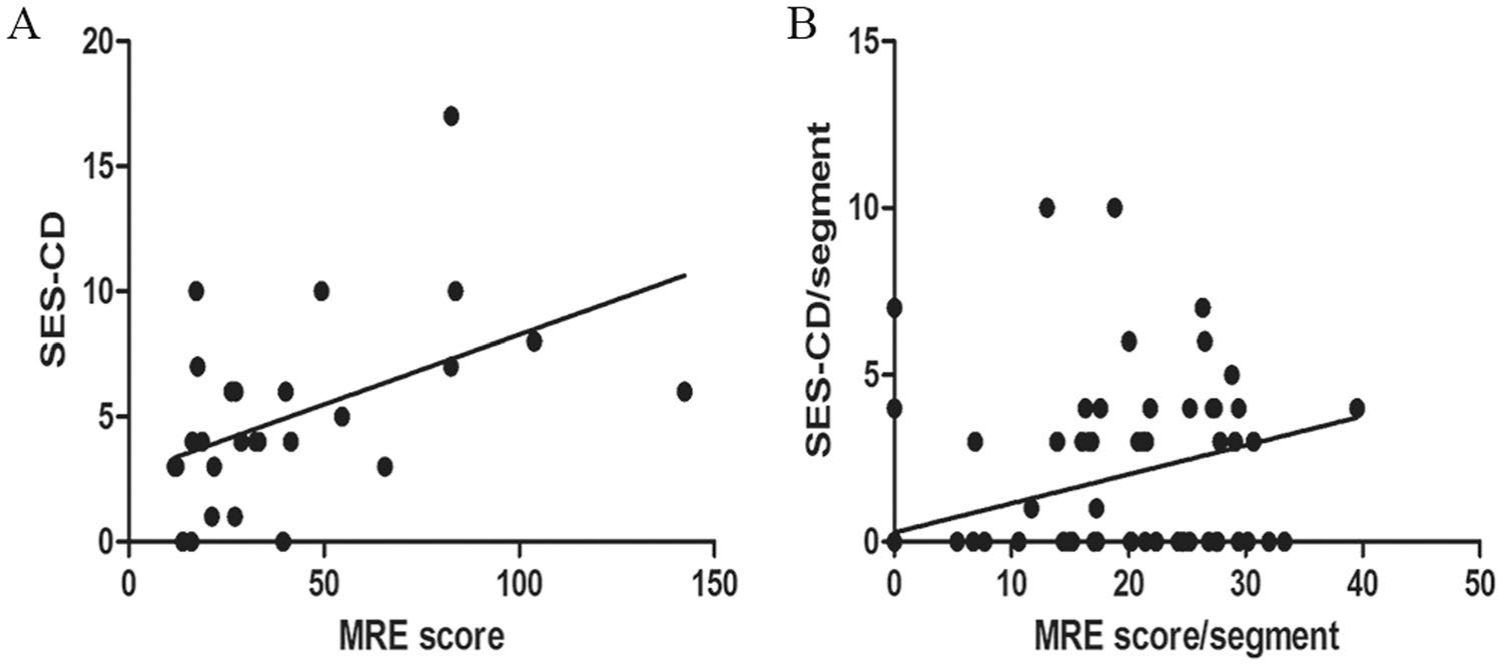
(**A**) Scatterplot demonstrating the correlation between Magnetic Resonance Enterocolonography (MRE) score and the Simple Endoscopic Score for Crohn’s disease (SES-CD). The Spearman rank correlation coefficient was r = 0.527 (P = 0.005, n = 27); (**B**) Scatterplot demonstrating the correlation between the MRE score/segment and the SES-CD/segment. The Spearman’s rank correlation coefficient was r = 0.590 (P < 0.001, n = 135).

To calculate the agreement between MaRIA and SES-CD scores, we summarized the results for patients who underwent both MRE and enteroscopy during a single hospitalization. This approach meant that the interval time between the two examinations was no more than 7 days. A total of 27 patients were included in the analysis. Enteroscopy and MRE were performed on the same day for 11 patients. The median SES-CD was 4.0 (range 3.0–7.0), while the median MaRIA was 28.8 (range 17.6–54.7). There was moderate agreement between the SES-CD and MaRIA results (r = 0.527, P = 0.005, n = 27, Fig. 1A). Considering that CD lesions typically follow a skip pattern, segment-by-segment analysis was undertaken to further evaluate the correlation between MRE changes and endoscopic findings. A significant correlation between the two scores was found (r = 0.590, P < 0.001, n = 135, Fig. 1B).

**Figure 2:**
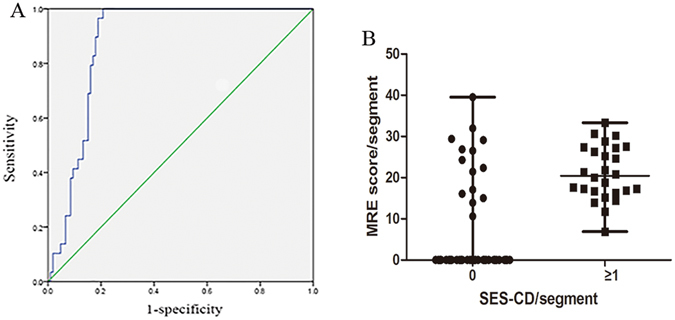
(**A**) Receiver operating characteristic (ROC) curve of MRE score for each segment in predicting endoscopic mucosal healing. The area under the curve was 0.881 (95% confidence interval (CI): 0.825–0.937); (**B**) MRE score/segment in the endoscopic mucosal healing group (SES-CD = 0) and the active mucosa group (SES-CD ≥ 1). The horizontal line in the middle is the median, and the lower line represents the lower quartiles, while the upper line represents the upper quartiles.

With regard to the assessment of active mucosal lesions, the ROC analysis was further undertaken. When the endoscopic mucosal healing of each segment was defined as SES-CD = 0, MRE exhibited a good performance (area under the receiver operating characteristic curve (AUC): 0.881; 95% confidence interval (CI): 0.825–0.937, Fig. 2A). A cut-off value of 6.8 provided a sensitivity of 100%, a specificity of 79.2%, a positive predictive value (PPV) of 60%, and a negative predictive value (NPV) of 92.2% for detecting intestinal inflammation. The corresponding MaRIA in each segment categorized as the inactive and active group were shown in Fig. 2B.

### Correlations between FC with MRE and enteroscopy findings

FC level and MRE parameters from 29 patients were eligible for Spearman’s rank correlation analysis when classifying patients using the same aforementioned criteria. However, the levels of FC showed no correlation with the MaRIA (r = 0.230, P = 0.230). Different from the FC level, the CRP level exhibited a significant correlation with the MaRIA (r = 0.534, P = 0.003), followed by the ESR (r = 0.525, P = 0.003) and CDAI (r = 0.421, P = 0.023).

In the study, 109 patients whose FC levels and peripheral blood markers were measured within 1 week of enteroscopy were included. SBE was performed in 35 patients, and the median reach for the scope was 210 cm from the ileocaecal valve or the post-operative anastomosis. Seven out of 74 patients experiencing ICS had a major stenosis that could not be passed, and there were 4 patients in whom the SBE could not be inserted sufficiently to assess for proximal ileum lesions. In contrast, MRE could reach all the lesions sufficiently in each segment.

**Figure 3:**
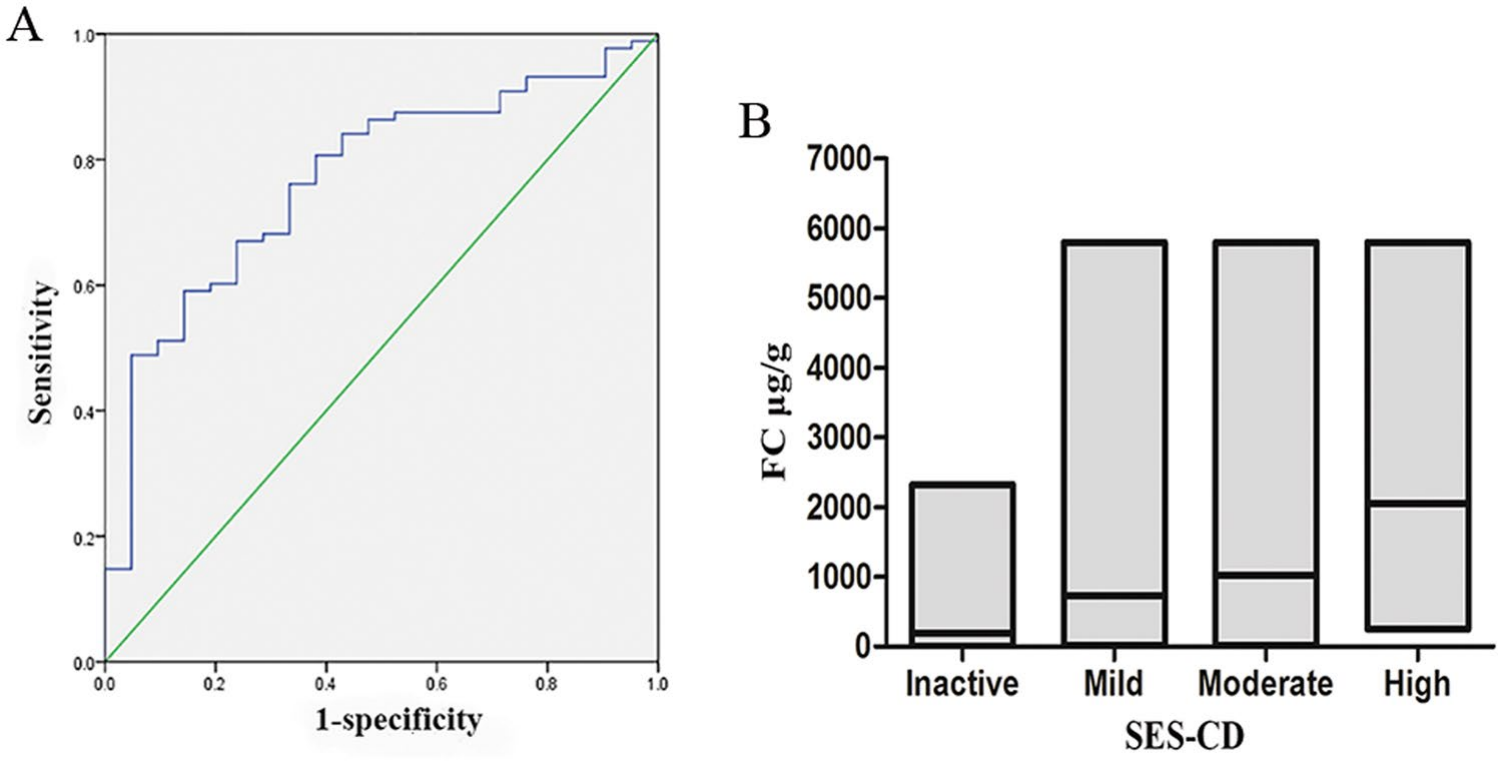
(**A**) Receiver operating characteristic (ROC) curve of faecal calprotectin in predicting endoscopic mucosal healing. The area under the curve was 0.768 (95% CI: 0.664–0.872); (**B**) Faecal calprotectin concentrations in different levels of endoscopic Crohn’s disease activity. The horizontal line in the middle of the box is the median, and the box represents the lower and upper quartiles.

The agreement between the FC level, ESR, CRP level, CDAI and SES-CD was calculated. SES-CD scores showed better agreement with FC levels (r = 0.503, P < 0.001) than with the ESR (r = 0.377, P < 0.001), CRP levels (r = 0.461, P < 0.001) or CDAI (r = 0.320, P < 0.01). Moreover, the AUC of the FC levels for endoscopic mucosal healing was 0.768 (95% CI (0.664–0.872), P < 0.001, Fig. 3A). And the FC cut-off value was 213.1 μg/g, with 76.1% sensitivity and 66.7% specificity and 90.5% PPV and a 40% NPV. Even in patients in whom the CD lesions were only in the small bowel, FC levels also demonstrated a strong relationship with mucosal healing (AUC 0.753, 95% CI (0.557–0.950), P = 0.035). A cut-off value of 170.2 μg/g provided a sensitivity of 80%, a specificity of 70%, a PPV of 80% and a NPV of 70%.

As summarized in Table 3, patients were divided into four subgroups according to SES-CD scores. Except for the CDAI, the other three biomarkers were significantly different among the subgroups. However, only the median levels of FC were sequentially elevated with the severity of the mucosal lesions, which is illustrated in Fig. 3B. Both the median ESR results and CRP levels in the high group were slightly lower than in the moderate group.

**Table 3:** Levels of FC, ESR, CRP and CDAI, sub-grouped according to SES-CD.

Endoscopic activity	Inactive	Mild	Moderate	High	P value
	(0–3)	(4–10)	(11–19)	(≥20)	
Number of patients	39	51	14	5	
Number of patients FC (μg/g)	39	51	14	5	<0.001
	197.3 (99.7–5628)	730 (254.4–1110.1)	1017.2 (488.9–1455.3)	2055.7 (818.2–4038.5)	
Number of patients ESR (mm/h)	35	46	13	5	<0.001
	12 (6–23)	17.5 (11.0–37.5)	44 (34–76)	28 (12–104)	
Number of patients CRP (mg/L)	39	51	14	5	<0.001
	4.9 (1.7–25.3)	19.6 (9.1–47.7)	49.3 (12.0–60.9)	47.8 (27.5–125.6)	
Number of patients CDAI	37	47	14	5	0.052
	126 (68–217)	175 (107–233)	253 (113.2–330.0)	350 (108–489.5)	

Median interquartile range (IQR) for continuous variables; SES-CD, simple endoscopic score for Crohn’s disease; FC, faecal calprotectin; ESR, erythrocyte sedimentation rate; CRP, C-reactive protein; CDAI, Crohn’s disease activity index.

**Figure 4:**
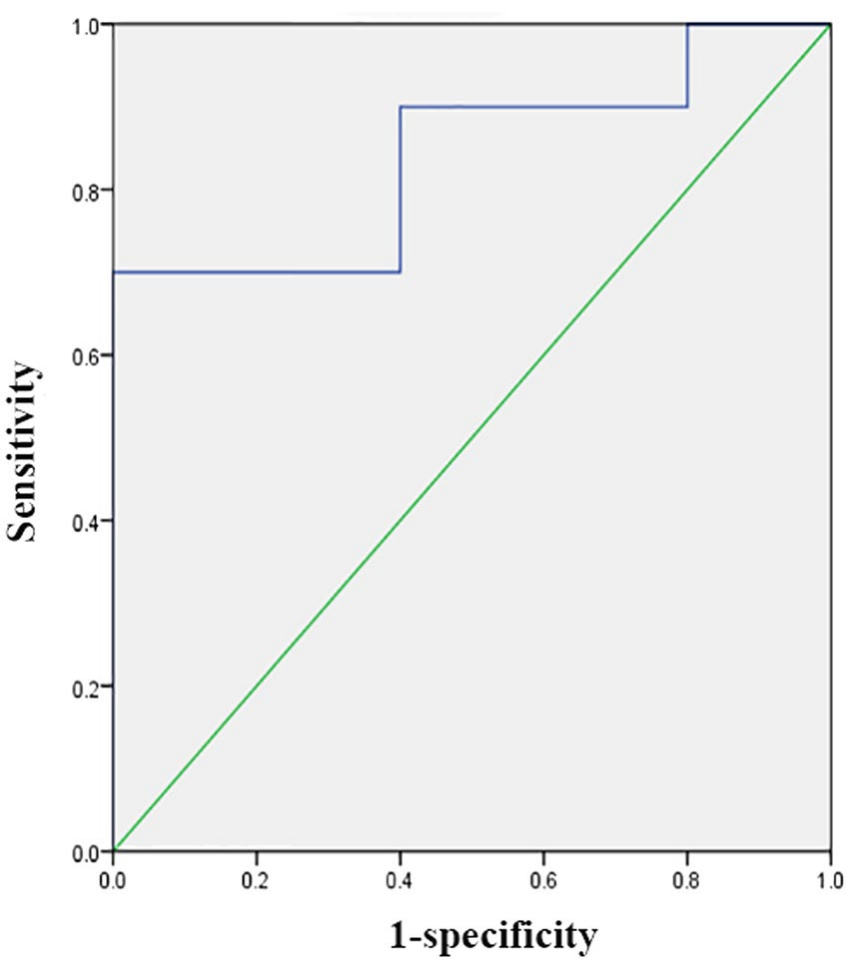
Receiver operating characteristic (ROC) curve of a combination of MRE score and faecal calprotectin and erythrocyte sedimentation rate and C-reactive protein and Crohn’s disease activity index in predicting endoscopic mucosal healing. The area under the curve was 0.840 (95% CI: 0.635–1.000).

Among MaRIA, FC, CRP, CDAI and ESR, multivariate analysis revealed that MaRIA was the only independent predictor for monitoring mucosal severity of patients, the OR(odds ratio) was 1.068 (95% CI: 0.974–1.171). Combined all the five parameters together, ROC analysis showed that the AUC for endoscopic mucosal healing was 0.840, 95% CI (0.635–1.000), P = 0.037 (Fig. 4).

## Discussion

In the treatment of CD, restaging is frequently required to monitor disease activity, which adds great economic burden and a risk of unexpected complications for patients^25^. The results of this study demonstrated that both FC levels and MRE scores provided clinically relevant data on small bowel CD. Non-invasive procedures would be more easily tolerated by patients compared with complicated and painful enteroscopy.

No correlation between FC levels and MRE findings was observed in our study. However, Elena *et al*. reported that FC levels were significantly correlated with the degree of MRE inflammatory activity (Spearman’s r = 0.56, P < 0.001)^26^. One possible explanation for the discrepancy is that a relatively small number of patients was eligible in the subgroup analysis (n = 29). To date, the MaRIA is the only validated MR scoring system for CD, and studies investigating the usefulness of the MaRIA to detect CD lesions of the ileal mucosa are scarce^12,27^. Kento *et al*. have evaluated the relationship between MRE score and SES-CD results in a prospective study of 125 ileal CD patients, and a close correlation was found (P < 0.001)^28^. Similar to their findings, we also reported a moderate correlation between the two scoring systems (r = 0.527, P = 0.005). However, most patients in their study were in clinical remission. In comparison, there were 14 out of 27 patients in clinical remission (defined as a CDAI lower than 150) and 13 patients with clinically active disease in our study. Hence, though there were a limited number of CD patients, the results may better reflect the findings in all types of CD patients. That a MaRIA score of higher than 6.8 in each segment had a high diagnostic accuracy for active mucosal lesions was consistent with previous studies^12,28^.

Several groups have shown that FC levels correlate with endoscopic disease activity in CD patients^5,29,30^, but few studies have assessed the correlation between FC levels and mucosal lesions in the deep small intestine in CD. In the present study, a moderate agreement was found between FC levels and small bowel CD. The cut-off value of 170.2 μg/g provided a relative high sensitivity and specificity for active disease in the small bowel. The CRP level is considered a cost-efficient biomarker of disease activity. However, in our study, FC levels correlated more closely with SES-CD result than CRP levels. Moreover, FC concentrations increased in parallel with the severity of mucosal inflammation, while the median CRP level failed to increase in cases of high endoscopic activity. This phenomenon could be attributed to a genetic heterogeneity of CRP response^31^. Thus, FC level is more sensitive for detecting the degree of endoscopic severity.

Recently, the Lemann score was proposed to measure the cumulative bowel damage at a specific point in time for CD patients^32^. The score is based on a comprehensive assessment of structural bowel damage, which requires a series of examinations including endoscopy and the history of surgical resection^32^. Though the score could be more comprehensive, it is more complicated that requires several steps. To date, there is no validated formula to calculate the global damage severity for the whole digestive tract, which greatly hampers its wide application in clinics. Therefore, to help patients release from pains as much as possible, our study was designed to evaluate the usefulness of MRE and FC in ileal CD. We found that the AUC could reach 0.84 when combined biomarkers and MaRIA and FC, while MaRIA alone could be the independent factor detecting mucosal inflammation.

There are some limitations to our study. The most relevant is that it is a single centre retrospective study with a limited number of patients who underwent endoscopy and MRE within one week. A multicentre study is warranted to further confirm our findings. Then, not all patients experienced SBE to evaluate for small bowel lesions, and it is known that ICS is inadequate to find CD lesions beyond the terminal ileum. Finally, the sensitivity and specificity of MRE for oedema and ulceration in the rectal segment are lower than for active lesions in other segments such as the colon, which has also been reported in a previous study^24^.

In summary, the FC level and MRE could provide accurate information on small bowel CD. When combined, the FC level and MRE represent a reliable alternative to painful endoscopy and can save patients from radiation exposure. Our work needs to be validated in larger, multicentre studies.

## Electronic supplementary material

Figure S1
