# Author Correction: Levels of Faecal Calprotectin and Magnetic Resonance Enterocolonography Correlate with Severity of Small Bowel Crohn’s Disease: A Retrospective Cohort Study

**DOI:** 10.1038/s41598-018-23086-y

**Published:** 2018-03-19

**Authors:** Lei Ye, Wei Cheng, Bi-qin Chen, Xing Lan, Shao-dong Wang, Xiao-chen Wu, Wei Huang, Fang-yu Wang

**Affiliations:** 1 Department of Gastroenterology and Hepatology, Jinling Hospital, Medical School of Nanjing University, 305 Zhongshan East Road, Nanjing, 210002 Jiangsu Province China; 2 Department of Gastroenterology and Hepatology, Jinling Hospital, Clinical Medical School of Southern Medical University, Nanjing, 210002 China; 3 School of Medical Imaging, Xuzhou Medical University, Xuzhou, 221004 China; 4 Department of Medical Imaging, Jinling Hospital, Medical School of Nanjing University, 305 Zhongshan East Road, Nanjing, 210002 Jiangsu Province China

Correction to: *Scientific Reports* 10.1038/s41598-017-02111-6, published online 16 May 2017

The original version of this Article contained a typographical error in the spelling of the author Wei Cheng, which was incorrectly given as Wei Chen. This has now been corrected in the PDF and HTML versions of the Article.

