# Supplementary material for: Levels of Faecal Calprotectin and Magnetic Resonance Enterocolonography Correlate with Severity of Small Bowel Crohn’s Disease: A Retrospective Cohort Study
Source: Sci Rep. 2017 May 16;7:1970. doi: 10.1038/s41598-017-02111-6 (PMC5434057; doi:10.1038/s41598-017-02111-6)
Supplement: Supplementary file 1 — Figure S1 [file 41598_2017_2111_MOESM1_ESM.pdf]

Levels of Faecal Calprotectin and Magnetic Resonance Enterocolonography Correlate with Severity of Small Bowel Crohn's Disease: A Retrospective Cohort Study

Lei Ye[1,a], PhD, Wei Chen[1,a], MD, Bi-qin Chen[2,a],MD, Xing Lan[3], MD, Shao-dong Wang[1], PhD, Xiao-chen Wu[1], MD, Wei Huang[4], PhD, Fang-yu Wang[1,*], PhD

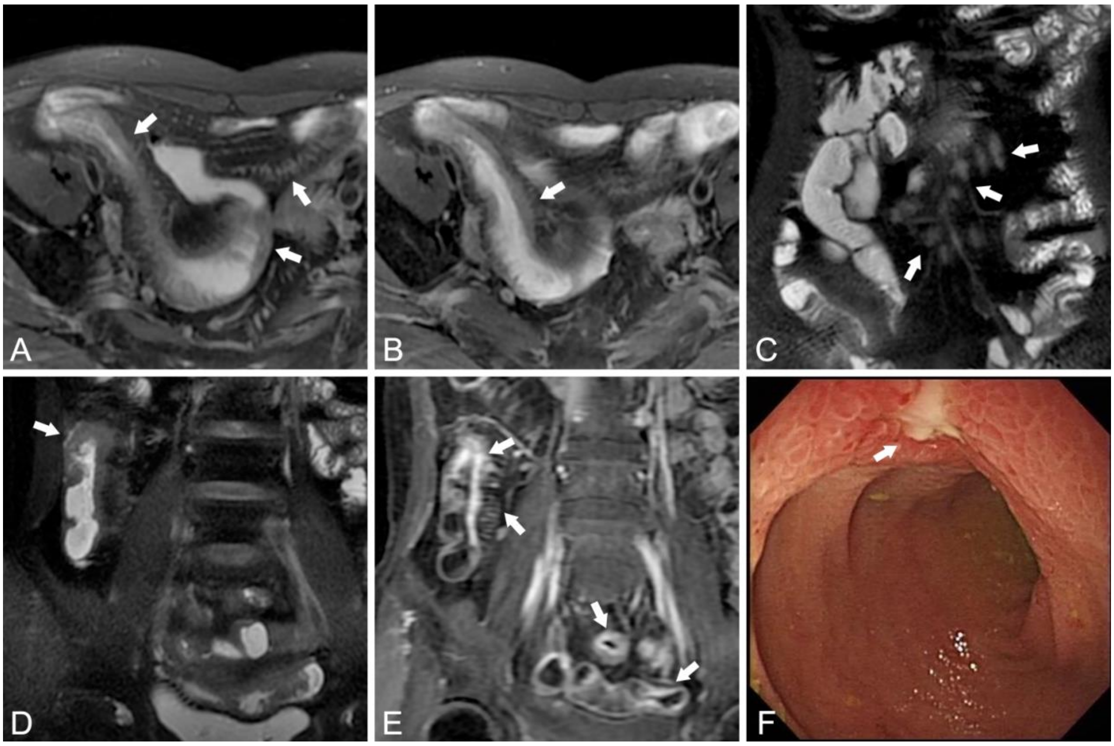

Figure S1 Conventional MRE findings in the distal ileum of a patient with small bowel CD. (A, B**)** Magnetic resonance axial T2-weighted images depicts mural thickening and moderate increase in signal-bowel wall of the distal ileum(arrow), indicating oedema in wall, and the presence of increased mesenteric vascularity(the "comb" sign). The mesenteric (arrow) shows multiple lymph nodes (C) and perimural tissue (arrow) shows a small rim of fluid (D). E, The affected bowl-wall shows marked enhancement (arrow) after intravenous administration. F, The enteroscopy

image of terminal ileum shows deep longitudinal ulcer (arrow).
